# Supplementary material for: Pulsed electroreduction of low-concentration nitrate to ammonia
Source: Nat Commun. 2023 Nov 14;14:7368. doi: 10.1038/s41467-023-43179-1 (PMC10645723; doi:10.1038/s41467-023-43179-1)
Supplement: Supplementary file 1 — Supplementary Information [file 41467_2023_43179_MOESM1_ESM.pdf]

Supporting Information

Pulsed Electroreduction of Low-concentration Nitrate to Ammonia

Yanmei Huang[1,2,7], Caihong He[3,7], Chuanqi Cheng[4], Shuhe Han[1], Meng He[1], Yuting Wang[1,2], Nannan Meng[1], Bin Zhang[1], Qipeng Lu[3,5,*], and Yifu Yu[1,2,6*]

[1]Institute of Molecular Plus, School of Science, Tianjin University, Tianjin 300072, China.

[2]Haihe Laboratory of Sustainable Chemical Transformations, Tianjin 300192, China

[3]School of Materials Science and Engineering, University of Science and Technology Beijing, Beijing 100083, China

[4]Institute of New Energy Materials, School of Materials Science and Engineering, Tianjin University, Tianjin 300072, China

[5]Shunde Innovation School, University of Science and Technology Beijing, Foshan 528399, China

[6]Tianjin University-Asia Silicon Joint Research Center of Ammonia-Hydrogen New Energy, Xining 810000, China

[7]These authors contributed equally: Yanmei Huang, Caihong He.

[*]E-mail: qipeng@ustb.edu.cn (Q.L); yyu@tju.edu.cn (Y.Y.)

Supplementary Table 1 Cell parameters of $RuIn_3$ determined from the XRD data in **Fig. 1a**.

$RuIn_3$	
Space group	P42/mnm
a	6.997 Å
b	6.997 Å
c	7.242 Å
α	90 °
β	90 °
γ	90 °

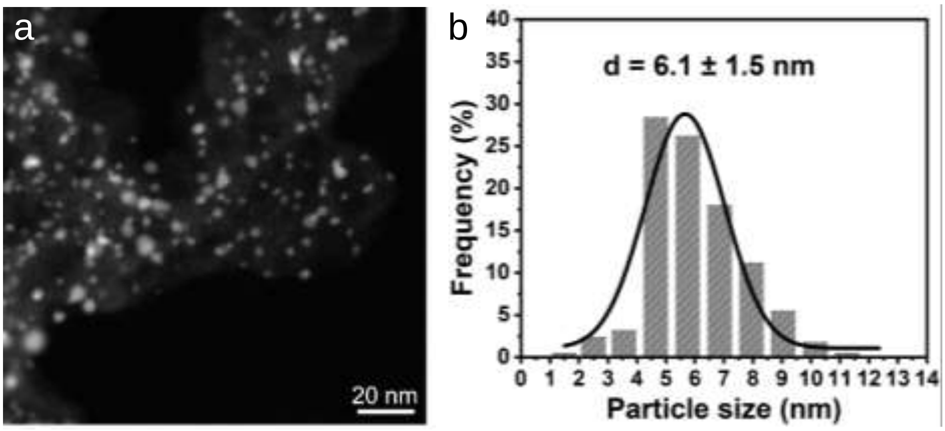

Supplementary Fig. 1 (a) HADDF-STEM image, and (b) the corresponding particle size distribution.

The HAADF-STEM image shows the small nanoparticles of $RuIn_3$ with an average size of ~6.1 nm deposited on the carbon black support.

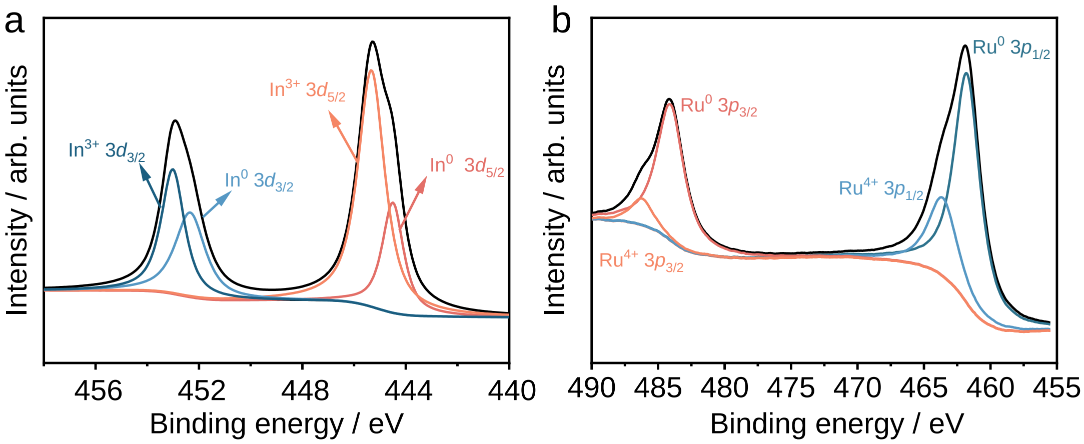

Supplementary Fig. 2 XPS spectra of $RuIn_3$/C powder. (a) In 3*d* region and (b) Ru 3*p* region.

The X-ray photoelectron spectroscopy (XPS) spectra show that Ru mainly stays in the metallic state, while the majority of In is in the oxidized state, which can be ascribed to the surface oxidation of low-coordinated superficial indium atoms when exposed to air[1,2].

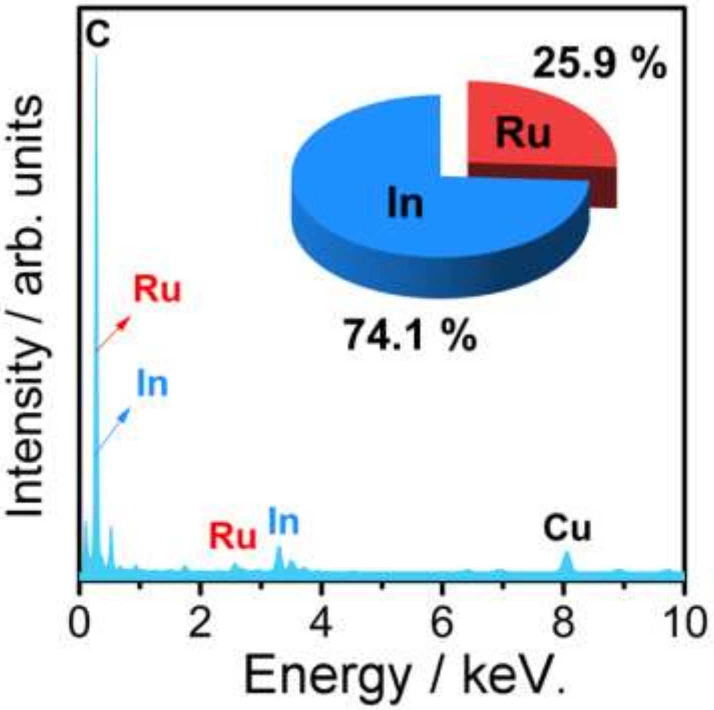

Supplementary Fig. 3 The EDX spectrum of the as-prepared $RuIn_3/C$.

According to the EDX spectrum of $RuIn_3/C$, the atomic ratio of Ru to In is 25.9:74.1, consistent with the ICP-OES result (Supplementary Table 2) and the phase composition obtained from XRD patterns.

Supplementary Table 2. ICP-OES results of as-prepared $RuIn_3/C$, Ru/C and In/C.

Sample	Ru (wt%)	In(wt%)	Ru/In (at %)
$RuIn_3/C$	12.08	42.36	11.95/36.89
Ru/C	11.73	\	\
In/C	\	39.25	\

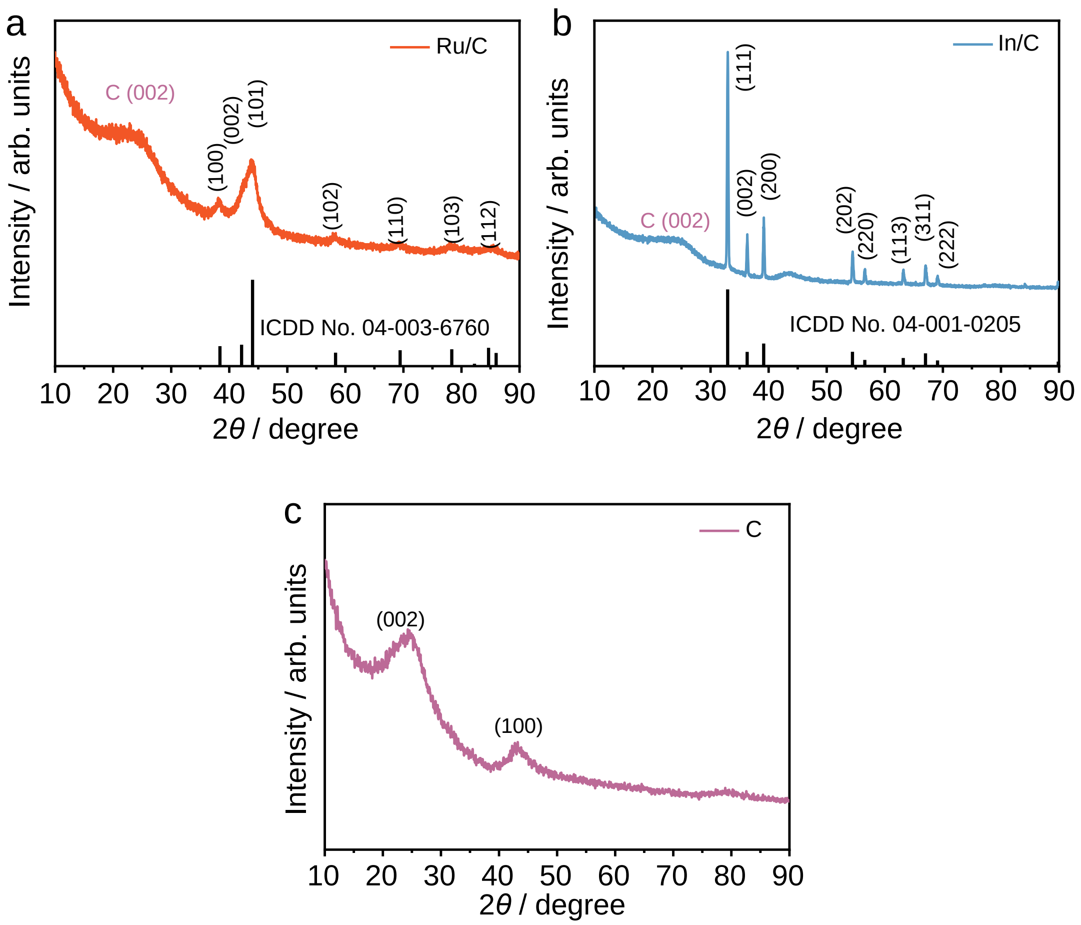

Supplementary Fig. 4 The XRD patterns of (a) Ru/C, (b) In/C, and (c) carbon substrate.

For comparison, carbon-supported Ru nanoparticles (Ru/C) and carbon-supported In nanoparticles (In/C) are prepared by the same synthesis methods as $RuIn_3/C$.

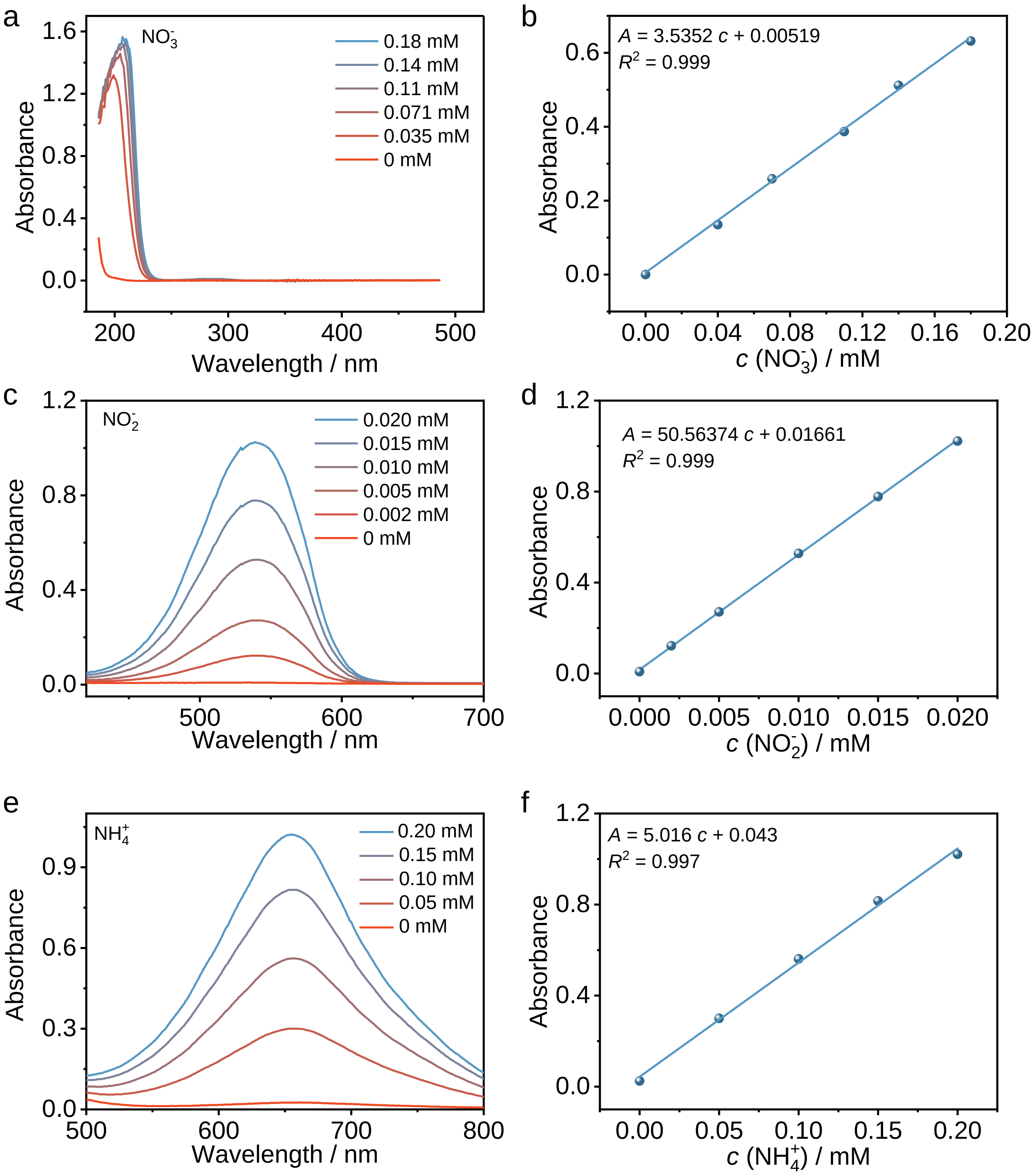

Supplementary Fig. 5 NO_3^-, NO_2^-, and NH_4^+ quantification using UV−vis absorption spectroscopy. (a) UV−vis absorption spectra and (b) the corresponding calibration curve for NO_3^-. (c) UV−vis adsorption spectra and (d) the corresponding calibration curve for NO_2^-. (e) UV−vis absorption spectra and (d) the corresponding calibration curve for NH_4^+.

The calibration curves of NO_3^-, $NO_2^{-,}$ and NH_4^+ all show good linearity relationship.

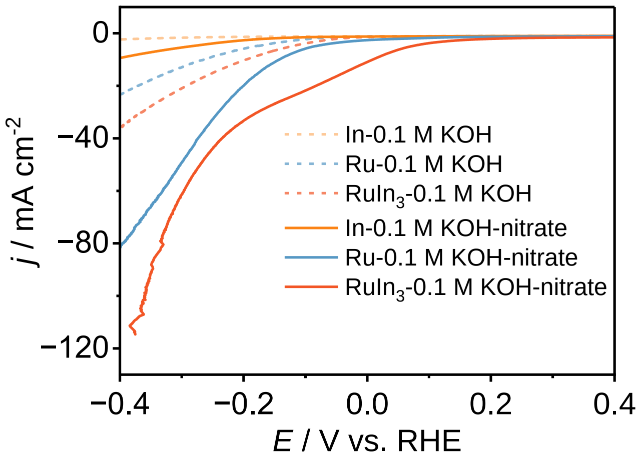

Supplementary Fig. 6 LSV curves of various catalysts in 0.1 M KOH solution with and without 10 mM nitrate under 1800 rpm with 85% iR correction.

For Ru/C and $RuIn_3$/C, the LSV curves show a noticeable increase in current density after adding 10 mM nitrate, indicating the happening of nitrate electroreduction.

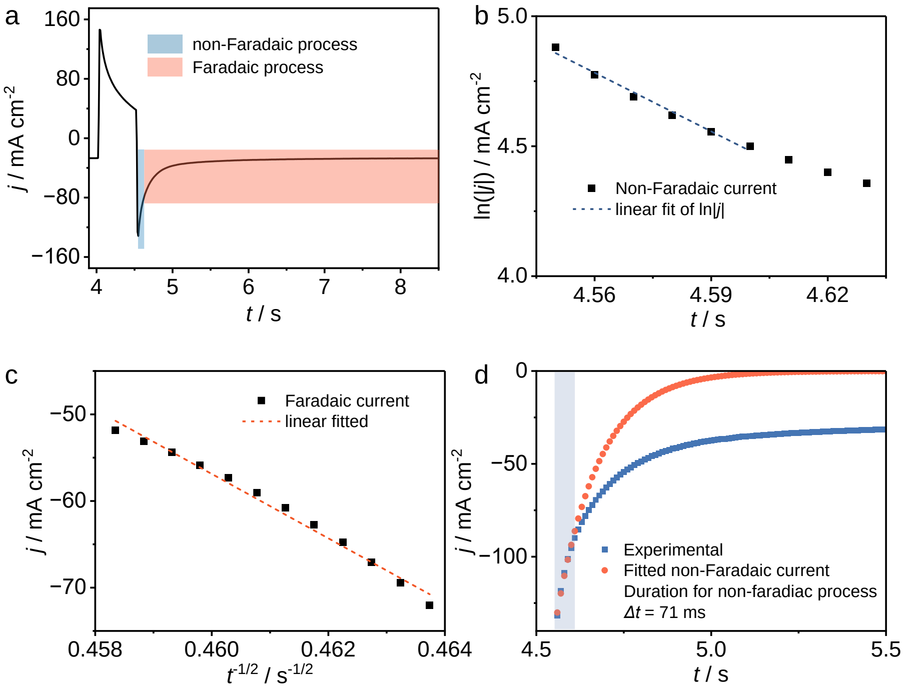

Supplementary Fig. 7 (a) Complete pulse profile of a 4.5 s pulse (t_a = 0.5 s, t_c = 4 s). (b) Natural log of the non-faradaic current from the blue inset with a linear fit. (c) The Faradaic current from the red inset with a linear fit was averaged over all pulses to determine the average faradaic current. (d) The fitted non-Faradaic current and the determined duration for the non-Faradaic process.

The transient current spikes were significant after the step change in potential (Supplementary Fig. 7a). This type of transient electrochemical current is commonly associated with a non-Faradaic process, usually due to a capacitive current arising from the charging or discharging of the electrical double-layer, or Helmholtz layer, at the electrode surface, rather than a Faradaic reaction current[3,4]. According to Kimura's study, the non-Faradaic process followed an I vs. e^{-t} relation (Supplementary Fig. 7b), while the Faradaic process followed an I vs. $t^{-1/2}$ relation (Supplementary Fig. 7c)[5]. Accordingly, the estimated time scale for the non-Faradaic process is approximately 71 ms (Supplementary Fig. 7d), which is in reasonable agreement with the value estimated by Hanrath et al., <0.1 s [5,6]. The residual of the cathodic time is used to reduce nitrate to ammonia.

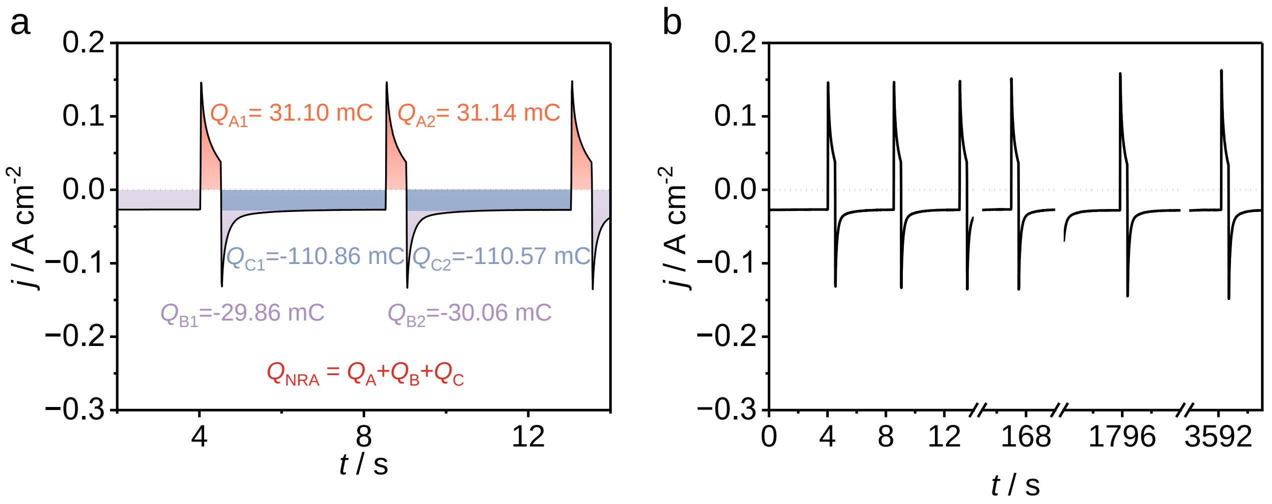

Supplementary Fig. 8 (a) The typical *i-t* curve of a pulsed NRA experiment. (b) Example of a current transient of pulsed NRA at $t_c = 4$ s and $t_a = 0.5$ s with the corresponding oxidative (Q_A), reductive (Q_B) and NRA charges (Q_{NRA}).

It is known that charge is needed to calculate the corresponding Faradaic efficiencies, which are calculated as the integral of the current over time. Thus, we integrated each cathodic pulse current and summed them. However, the current under pulsed conditions is not totally Faradaic but also contains contributions of i) capacitive changes due to the potential changes and ii) surface oxidation and reduction.[7] These additional contributions are difficult to quantify and lead to an overall error estimation, which is different for each number of created oxides and for different pulse sequences. The error is overcome by assuming that i) the capacitive charges are the same for each anodic and its subsequent cathodic pulse and ii) the current contribution from the oxides created at the anodic pulse corresponds to the current of their reduction.[7] This is a valid approximation because currents in the Q_A and Q_B regions should only reflect processes that are likely reversible (Supplementary Fig. 8a). Even at the end of one pulse sequence, the reversible trend remained (Supplementary Fig. 8b). The mathematical integration of the whole current transient, including the anodic charge, is therefore equivalent to the integration of each pulse with correction for the non-Faradaic contributions included. This simplification allowed us to better compare the different pulse lengths and the different amounts of oxides created. This assumption method has also been adopted in the reported literature[7-10].

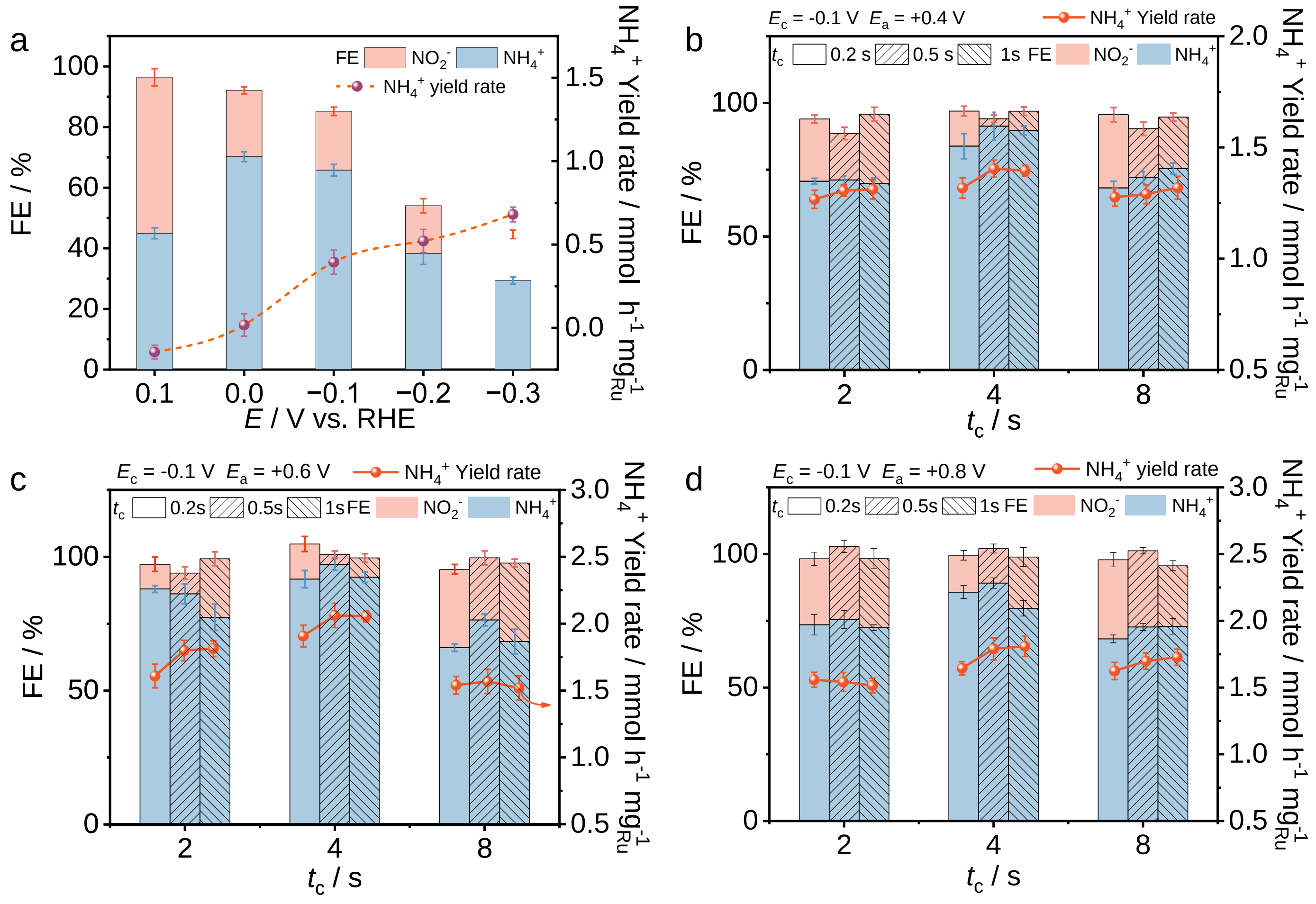

Supplementary Fig. 9 Screening of optimal pulse parameters. (a) Potentiostatic conditions with different applied potentials. Screening of different anodic and cathodic times at (b) E_a = 0.4 V, (c) E_a = 0.6 V, (d) E_a = 0.8 V.

With the negative shift of the applied potential under constant potential conditions (from 0.1 V to -0.3 V), the ammonia Faradaic efficiencies display a volcano shape, while the ammonia yield rate continuously increases. Thus, −0.1 V is chosen as E_c for the following parameter screening under pulse conditions. With the ammonia Faradaic efficiency and yield rate as the evaluator index, E_a = 0.6 V, t_a = 0.5 s, and t_c = 4 s are selected as the basic parameters for the following pulse-related experiments and characterizations.

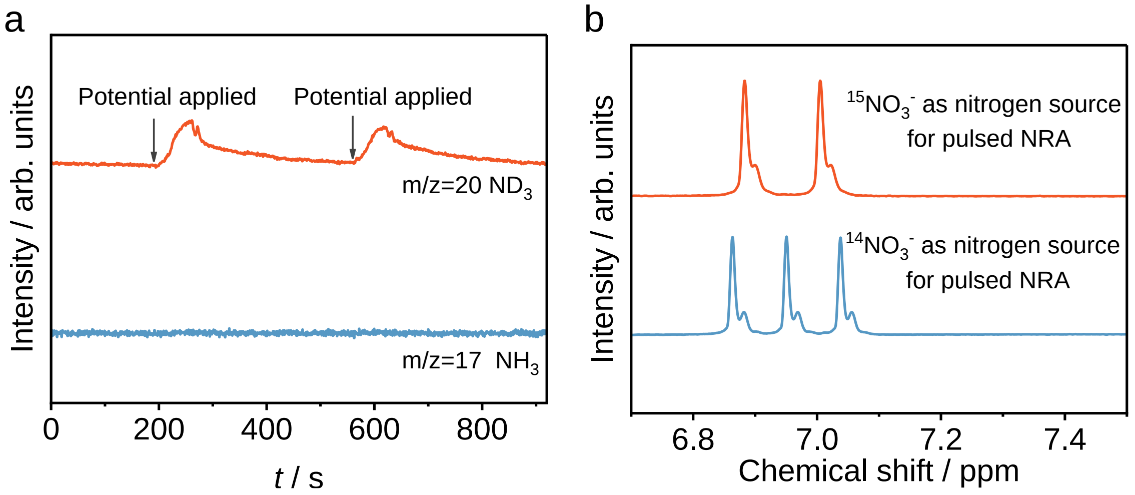

Supplementary Fig. 10 (a) The D isotope labelling online DEMS experiment and (b) ^{15}N-isotope labelling experiments under pulsed conditions (E_a = +0.6 V, t_a = 0.5 s, E_c = -0.1 V, t_c = 4 s) over $RuIn_3$/C.

Online differential electrochemical mass spectroscopy (DEMS) experiments were carried out using 0.01 M nitrate + 0.1 M NaOD + D_2O as electrolyte. Only the signals of m/z = 20, assigned to the generation of ND_3, were detected (Supplementary Fig. 10a). These phenomena confirmed that dissociation water provided the hydrogen source for the hydrogenation of nitrate. Moreover, ^{15}N-isotope labelling experiments demonstrated that the nitrogen in the generated ammonia originated from nitrate instead of pollutants in the external environment (Supplementary Fig. 10b).

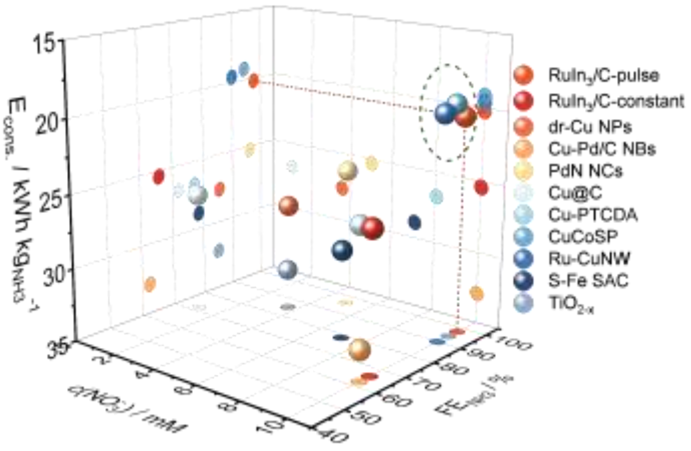

Supplementary Fig. 11 The NRA performance (c (NO_3^-) ≤ 10 mM) comparison between $RuIn_3$/C under pulsed conditions and the other reported catalysts under potentiostatic conditions.

The calculation of ammonia energy efficiency and electricity cost for producing 1 kg NH_3 per day from pulsed NRA was referenced to the previously reported literature[11,12].

The partial current (I_{NH_3}) for production 1 kg NH_3 was calculated as

$$I_{NH_3} = \frac{Q_{NH_3}}{t} = \frac{zFn_{NH_3}}{t} \quad \text{Supplementary Equation (1)}$$

Where z is the number of electrons transferred in nitrate reduction to ammonia. F is the Faraday constant. t is the time.

The total current needed is then given by diving by the Faradaic efficiency.

$$I_{total} = \frac{I_{NH_3}}{FE} \quad \text{Supplementary Equation (2)}$$

The required power is given from $P=UI$:

$$\text{power} = P = U \times I = \text{cell potential} \times I_{total} \quad \text{Supplementary Equation (3)}$$

$$U = \text{cell potential} = E_{OER} - E_{NRA} \quad \text{Supplementary Equation (4)}$$

We assumed that the overpotential of the water oxidation is zero and that there are no ohmic losses. Thus, E_{OER} is 1.23 V. E_{NRA} is the experimental value of the cathodic potential. The cell potential (E_{OER}- E_{NRA}) equals (1.23- E_{NRA}) V.

Given the specific characteristics of the pulse process and learning from Gu et al. studies[13], the effective cathodic energy consumption efficiency ($E_{cathodic\_consum}$) was considered.

$$E_{cathodic_{consum}} = \frac{|Q_{Cathodic}|}{|Q_{Cathodic}|+|Q_{anodic}|} \quad \text{Supplementary Equation (5)}$$

$Q_{cathodic}$ is the integral of the cathodic part current, and Q_{anodic} is the integral of the anodic part current. The effective cathodic energy efficiency at E_a = +0.6 V, t_a = 0.5 s, E_c = -0.1 V, and t_c = 4 s was calculated to be 82.8%. Some additional energy (about 17.2%) was used during the auxiliary oxidation or prereduction step.

The electricity power requirement is calculated from the power and the total time (1 day) after considering the energy waste.

$$\text{Electricity requirement} = \frac{\text{power}\times 24\text{ hour}}{\text{E}_{\text{cathodic}_{\text{consum}}}} \qquad \text{Supplementary Equation (6)}$$

The electricity cost is calculated from the electricity requirement and the price of electricity.

$$\text{Electricity cost} = \text{Electricity requirement} \times \text{price} \qquad \text{Supplementary Equation (7)}$$

The cost of electricity was calculated based on the price of renewable electricity alone (US$0.03 kWh^{-1})[14], the energy consumption and production cost of 1 kg NH_3 over $RuIn_3$/C through pulsed electrolysis were calculated as ~20.7 kWh and US$0.62. Note that this is a simple cost accounting based on electricity price without considering capital costs and ohmic losses. Considering the environmental benefit of nitrate contaminant removal, the reported $RuIn_3$/C for pulsed NRA is very appealing.

The ammonia energy efficiency was calculated with the following equation:

$$\text{EE}_{\text{NH}_3} = \frac{\left(E^0_{OER}-E^0_{NH_3}\right)}{E_{OER}-E_{NRA}} \times FE_{NH_3} \times \text{E}_{\text{cathodic}_{\text{consum}}} \qquad \text{Supplementary Equation (8)}$$

where E^0_{NH3} represents the equilibrium potential of nitrate electroreduction to ammonia, which was 0.69 V vs. RHE [11]. E^0_{OER} represents the equilibrium potential of the oxygen evolution reaction, which was 1.23 V vs. RHE. E_{NRA} is the experimental value of the cathodic potential. We assumed that the overpotential of the water oxidation is zero and that there are no ohmic losses. Thus, E_{OER} is 1.23 V, and the cell potential (E_{OER}- E_{NRA}) equals (1.23- E_{NRA}) V. FE_{NH3} is the Faradaic efficiency for ammonia.

Supplementary Table 3. The comparison of catalytic performance over recently reported electrocatalysts toward NO_3^- electroreduction at low concentrations (≤10 mM).

Power mode	Catalysts	Electrolyte	*E*/V vs. RHE	Faradaic efficiency / %	Energy efficiency / %	Energy consumption for 1 kg NH_3/ kWh	Ref
Pulsed	$RuIn_3/C$	0.1 M KOH+10 mM NO_3^-	-0.1	97.6	31.6	20.7	This work
Constant potential	$RuIn_3/C$	0.1 M KOH + 10 mM NO_3^-	-0.1	65.8	26.7	25.4	This work
	dr-Cu NPs	0.5 M K_2SO_4 + 3.57 mM NO_3^-	-0.64	85.47	24.7	27.6	15
	Cu-Pd/C NBs	0.1 M KOH + 10 mM NO_3^-	-0.4	62.3	20.6	32.9	16
	meso-PdN NCs	0.10 M Na_2SO_4 + 5.0 mM NO_3^-	-0.7	96.1	26.8	25.3	17
	Cu@C	1 M KOH + 1 mM NO_3^-	-0.3	72	25.4	26.8	18
	CuCl_BEF	0.5 M Na_2SO_4 + 7.14 mM NO_3^-	-1	44.7	10.8	62.8	19
	Cu-PTCDA	0.1 M PBS + 8.06 mM NO_3^-	-0.4	77	25.5	26.7	20
	CuCoSP	0.1 MKOH + 10 mM NO_3^-	-0.175	94.2	36.2	18.8	21
	Ru-CuNW	0.1 MKOH + 10 mM NO_3^-	-0.098	90	36.5	18.6	22
	S-modified Fe SAC	0.02 M Na_2SO_4 + 7.1 mM NO_3^-	-0.57	78.4	23.5	28.9	23
	TiO_{2-x}	0.5 M Na_2SO_4+7.1 mM NO_3^-	-0.94	85	21.1	32.2	24

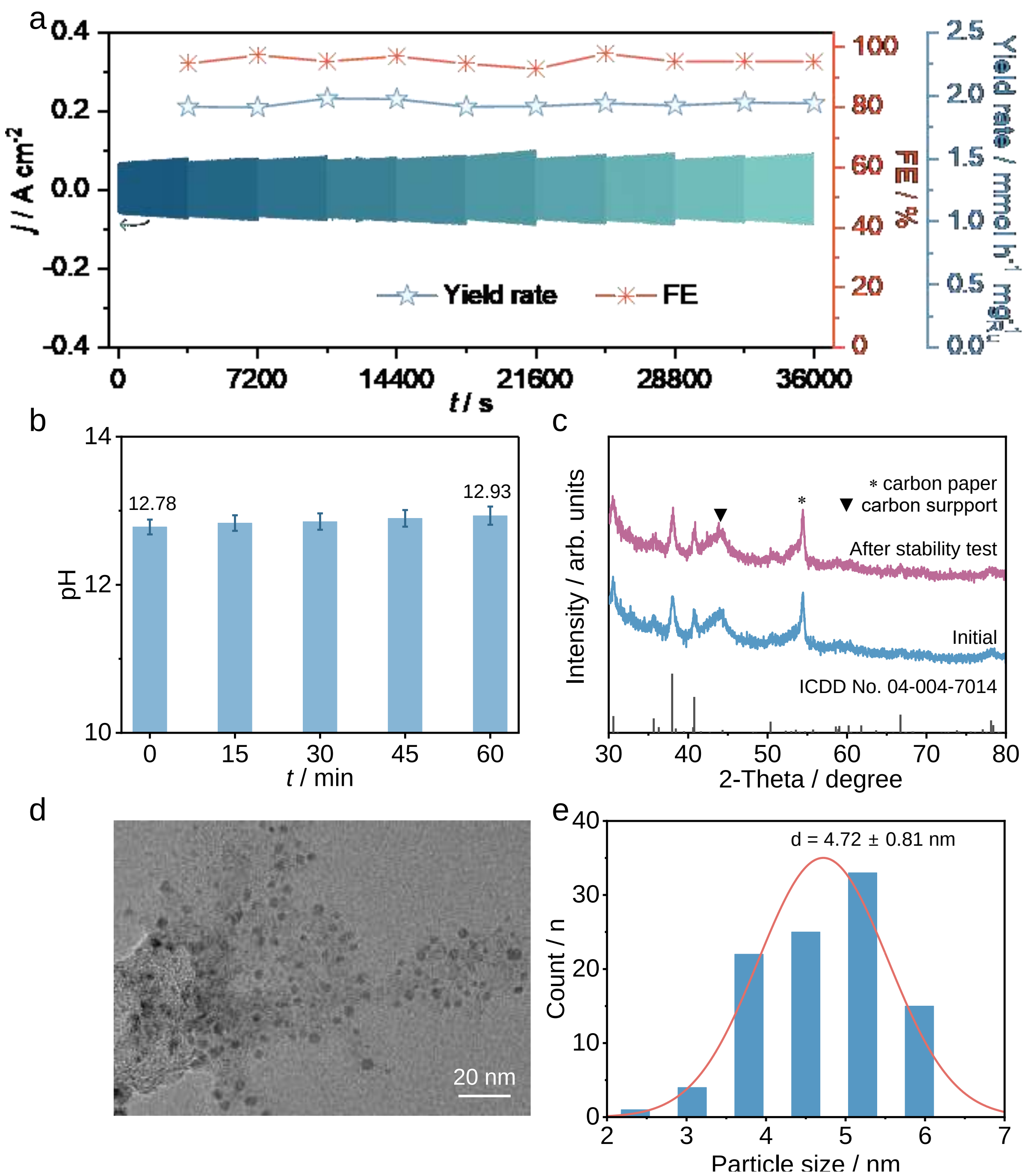

Supplementary Fig. 12 Cycle stability of pulsed NRA over $RuIn_3/C$. (a) Cyclic durability test of $RuIn_3/C$ for 10 mM nitrate electroreduction to ammonia under pulsed conditions (E_c = -0.1 V, E_a = +0.6 V, t_c = 4 s, t_a= 0.5 s). (b) The change of pH values during the electrocatalysis process. (c) The XRD pattern of $RuIn_3/C$ before and after the cycle stability test under pulsed conditions. (d) TEM image of the $RuIn_3/C$ catalysts after the stability tests, and (e) the corresponding particle size distribution.

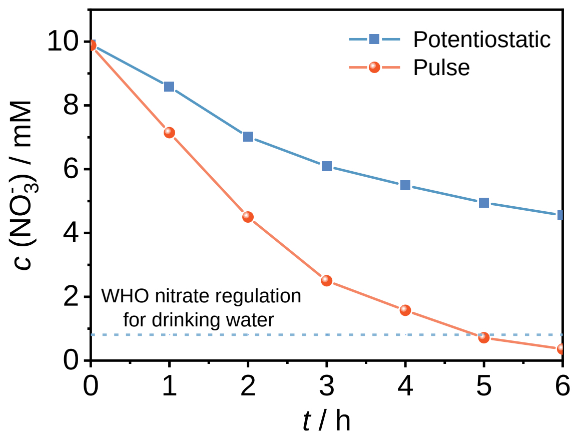

Supplementary Fig. 13 Time-dependent concentration change of NO_3^- over $RuIn_3/C$ under different conditions. At the potentiostatic condition, E = -0.1 V. At the pulse condition, E_a = 0.6 V, t_a = 0.5 s, E_c = -0.1 V t_c = 4 s.

After 5h pulsed electrolysis, nitrate concentration is reduced below the World Health Organization (WHO) regulations for drinking water[22,25]. For potentiostatic condition, the residual concentration is far from the emission standard.

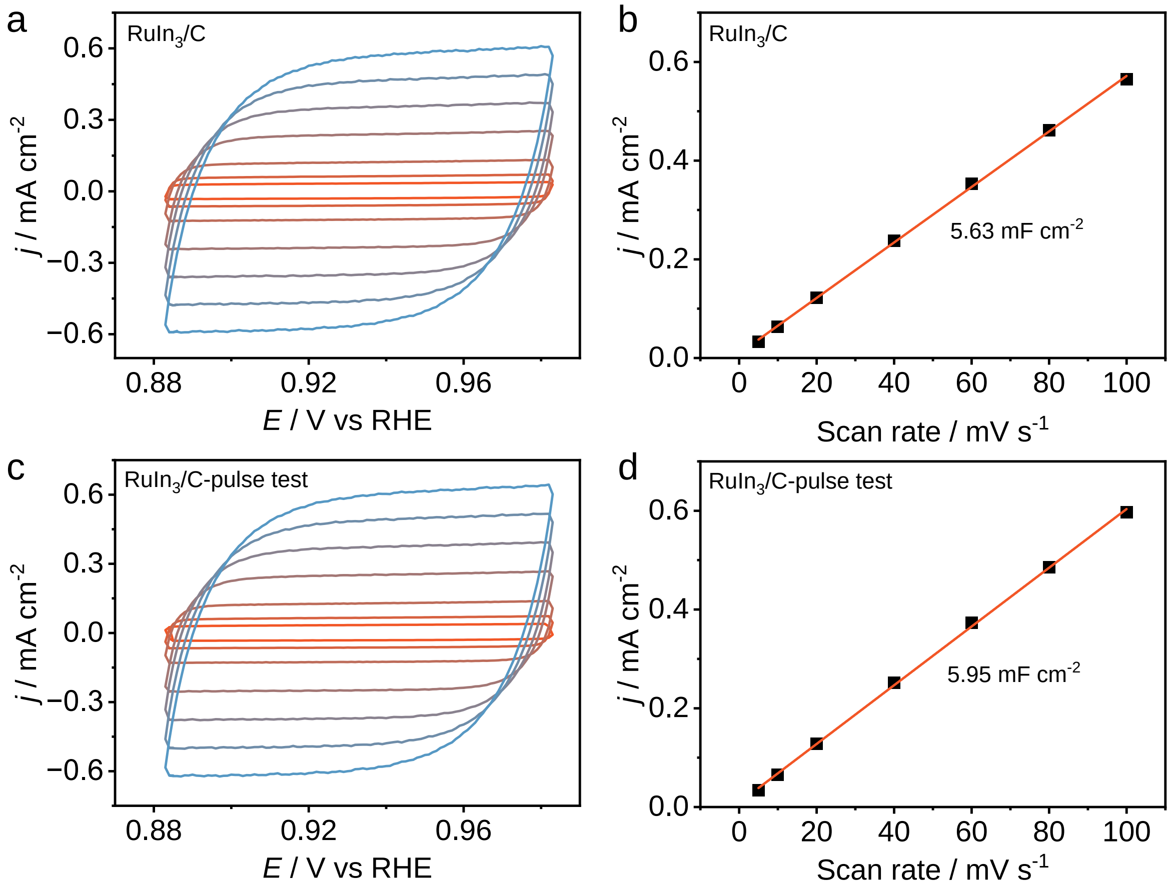

Supplementary Fig. 14 Calculation of double-layer capacitances of $RuIn_3$/C before and after pulsed test. (a) CV curves of $RuIn_3$/C and (b) the corresponding plots of the current density versus the scan rate. (c) CV curves of $RuIn_3$/C after pulse test and (d) the corresponding plots of the current density versus the scan rate.

The calculated double-layer capacitances before and after pulsed tests are 5.63 and 5.95 mF cm^{-2}, respectively, indicating that the surface reconstruction caused by the pulsed potential could be ignored.

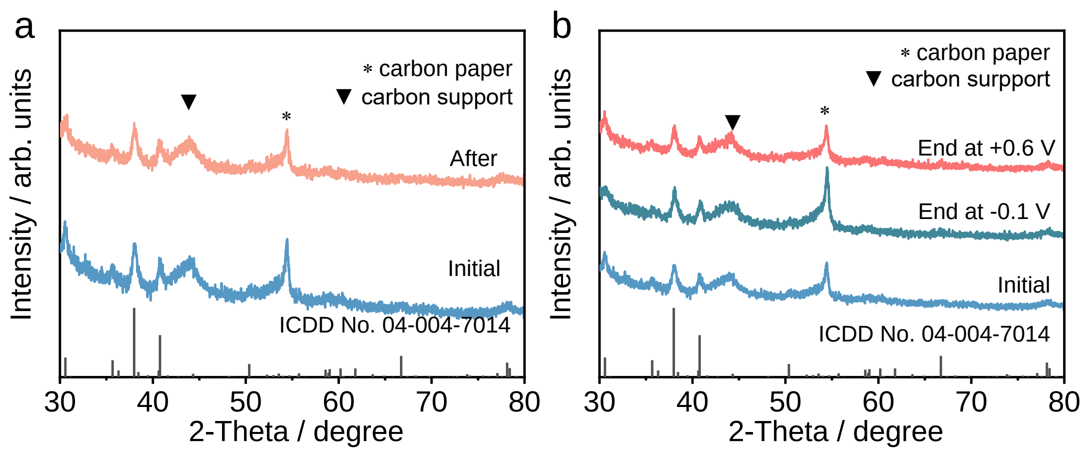

Supplementary Fig. 15 XRD patterns of carbon paper loaded $RuIn_3$/C with different treatments. (a) After potentiostatic electrolysis and (b) ending at different potentials.

The XRD patterns of carbon paper loaded $RuIn_3$/C with different treatments stay the same as the initial ones, indicating the well-maintained bulk crystal structure of $RuIn_3$ intermetallics.

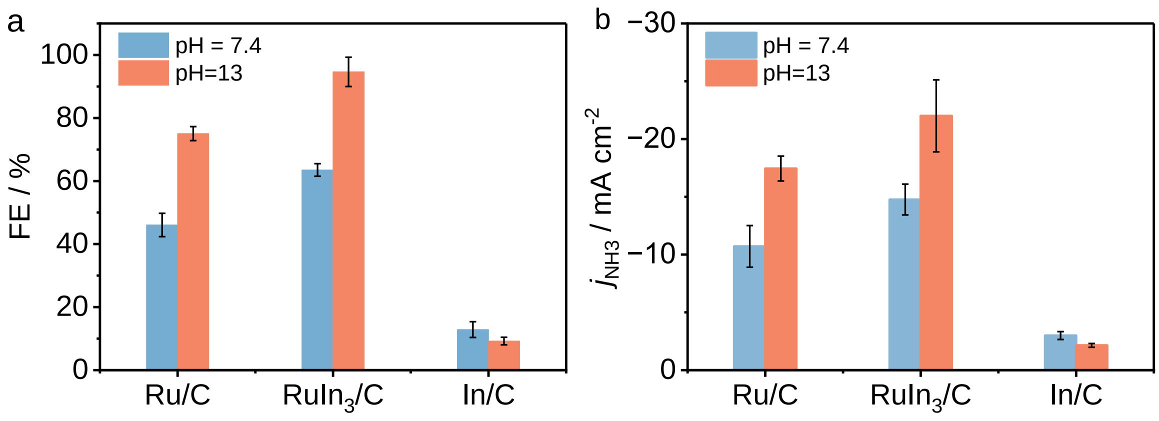

Supplementary Fig. 16 Performance comparison among different catalysts. The ammonia (a) Faradaic efficiency and (b) partial current density of $RuIn_3$/C, In/C, and Ru/C under pulsed conditions (E_a = 0.6 V, t_a= 0.5 s, E_c = −0.1, V t_c= 4 s) with different electrolyte pH. Note that 0.1 M phosphate buffer solution containing 10 mM nitrate was adopted as the electrolyte when considering the pH=7.4 condition.

Compared to $RuIn_3$/C and Ru/C, In/C shows ignorable activity for NRA under the same conditions (Supplementary Figs. 6 and 16), indicating the active species role of ruthenium.

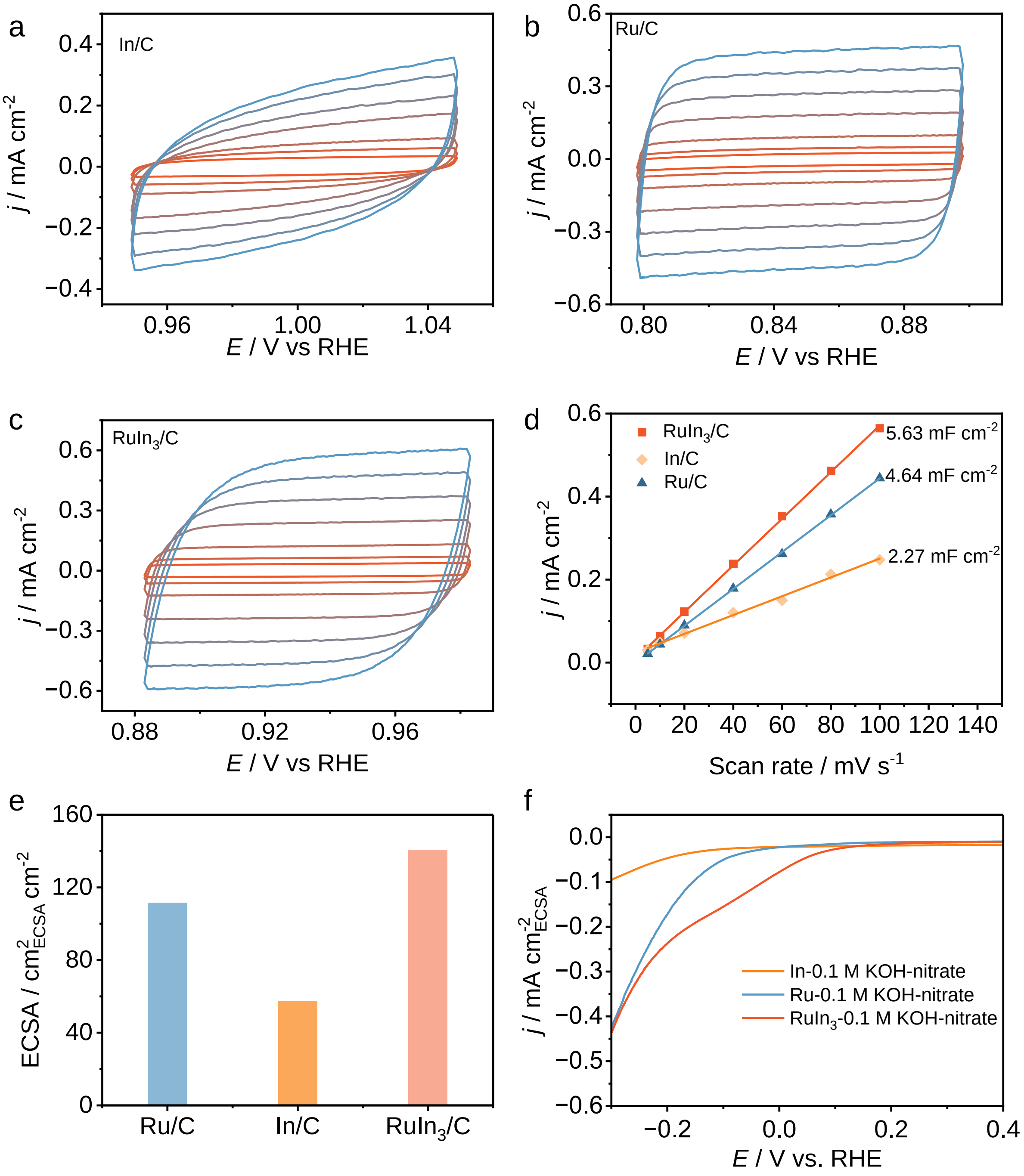

Supplementary Fig. 17 CV curves of (a) In/C, (b) Ru/C and (c) $RuIn_3$/C with various scan rates from 5 mV s^{-1} to 100 mV s^{-1}. (d) Fitting lines of the current density versus the different scan rates. (e) The calculated ECSA. (f) The LSV curves normalized to the ECSA for distinct catalysts.

The electric double-layer capacitance tests show that the ECSA of Ru/C, In/C, and $RuIn_3$/C is 57, 116, and 141 cm_{ECSA}^2 respectively (Supplementary Figs 17a-e). Notably, the LSV curve of $RuIn_3$/C after normalized to the ECSA still possesses the highest current density (Supplementary Fig 17f), indicating the highest intrinsic activity of $RuIn_3$/C.

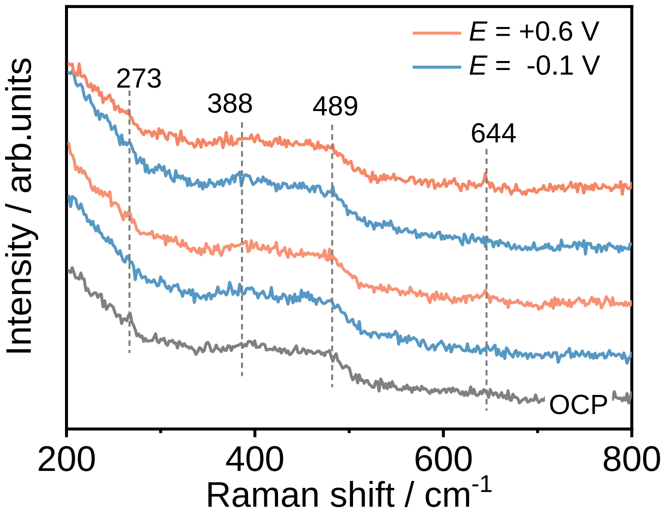

Supplementary Fig. 18 In situ Raman spectra of carbon paper loaded with $RuIn_3$/C at different potentials.

The first Raman scan without electricity is collected as the open circuit potential (OCP) spectrum. Then, the applied potential is set to -0.1 V and collects the spectra simultaneously. After that, the applied potential is switched to +0.6 V and collects the spectra simultaneously. This process is repeated twice. Only the bands at 273, 388, and 489 cm^{-1}, correlated to the stretching frequency for the In-O bond, can be observed at the OCP condition.[26,27] The observed In-O bonds at the OCP are related to the surface oxidation of low-coordinated superficial indium atoms when exposed to air. Indium is maintained in the oxide state under both +0.6 V (E_a) and -0.1 V (E_c) because of the low theoretical potential for In_2O_3/In conversion ($\varphi^{\theta}_{(In2O3/In)}$ = -1.034 V vs. SHE, pH=14)[2]. When the applied potential is switched to +0.6 V, the stretching mode of the Ru-O bond (644 cm^{-1})[28,29] appears.

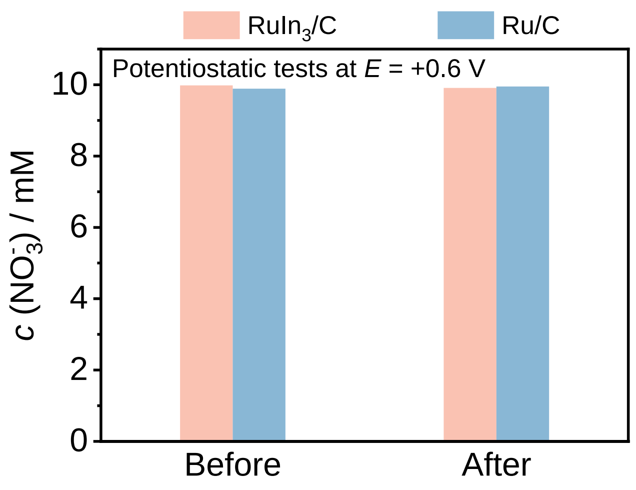

Supplementary Fig. 19 Nitrate concentration before and after the potentiostatic test at E = +0.6 V for 1 h.

$RuIn_3$/C and Ru/C exhibit no NRA performance under +0.6 V

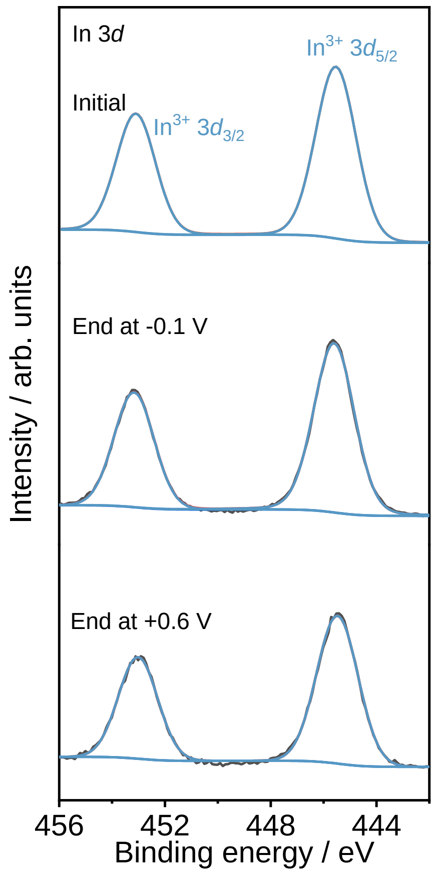

Supplementary Fig. 20 In 3*d* XPS spectra of $RuIn_3/C$ before pulse electrolysis and ending at different potentials.

Indium mainly exists in the oxide state before electrolysis, which can be ascribed to the smaller electronegativity of In (1.78) compared with that of Ru (2.20) and surface oxidation of low-coordinated superficial indium atoms when exposed to air[1,2]. Indium is maintained in the oxide state under both +0.6 V (E_a) and -0.1 V (E_c) because of the low theoretical potential for In_2O_3/In conversion ($\varphi^{\theta}_{(In2O3/In)}$ = -1.034 V vs. SHE, pH=14)[2].

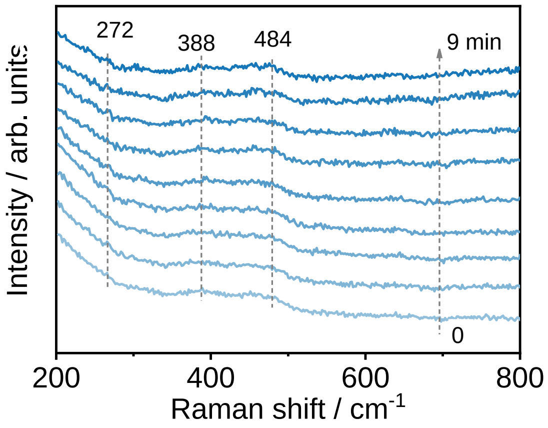

Supplementary Fig. 21 Time-dependent in situ Raman spectra collected during potentiostatic electrolysis at -0.1 V.

Under a constant potential at -0.1 V, with prolonged reaction time, the Raman bands arising from In_2O_3[26,27] remain almost the same as those in the initial state.

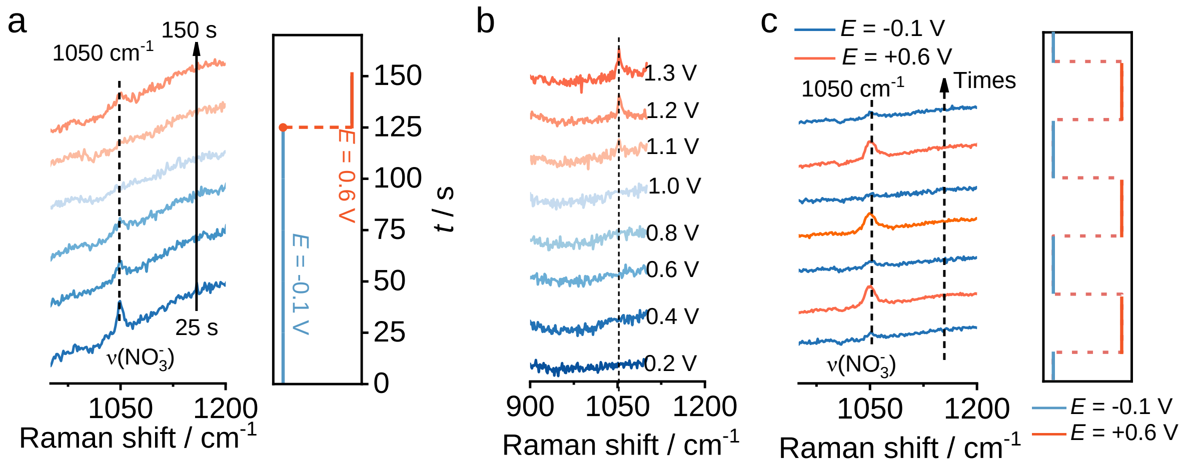

Supplementary Fig. 22 In situ Raman spectra over $RuIn_3/C$ collected under different conditions. (a) Raman spectra of $RuIn_3/C$ under -0.1 V for 125s and then switching to +0.6 V in 0.1 M KOH + 10 mM NO_3^- solution. (b) Raman spectra over $RuIn_3/C$ collected under different oxidation potentials in 0.1 M KOH+10 mM NH_4Cl solution. (c) Raman spectra of $RuIn_3/C$ under pulsed -0.1 V and then +0.6 V in 0.1 M KOH + 10 mM NO_3^- solution with 3 cycles.

As shown in Supplementary Fig. 22a, under −0.1 V, the characteristic peak of nitrate (~1050 cm^{-1})[21] gradually shrinks with the accumulation of scanning times and almost vanishes after 125 s of reduction. As the potential is switched to +0.6 V, the peak at ~1050 cm^{-1} emerges again, demonstrating nitrate accumulation near the positively charged electrode. To exclude the possibility that the accumulated NO_3^- originated from NH_3 oxidation, potential-dependent in situ Raman experiments are carried out. Only when the applied potential reaches 1.1 V the signal of nitrate can be observed in 0.1 M KOH+0.01 M NH_4Cl solution (Supplementary Fig. 22b). When the applied potential switched between −0.1 V and +0.6 V periodically, the normalized peak intensity of nitrate (~1050 cm^{-1}) exhibited a nearly periodic variation trend, in which the characteristic peak displayed a higher intensity at +0.6 V and then decreased to a relatively lower level at −0.1 V (Supplementary Fig. 22c).

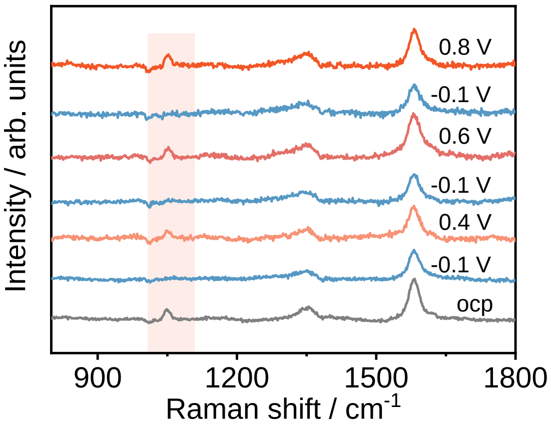

Supplementary Fig. 23 In situ Raman spectra of $RuIn_3/C$ begin at -0.1 V and then switched to different anodic potentials in 0.1 M KOH + 10 mM NO_3^- solution.

The nitrate accumulation effects under different E_a values are also considered. The peak intensity of nitrate at +0.6 V is almost the same as that at +0.8 V, indicating that +0.6 V is sufficient to renew the nitrate distribution near the electrode surface.

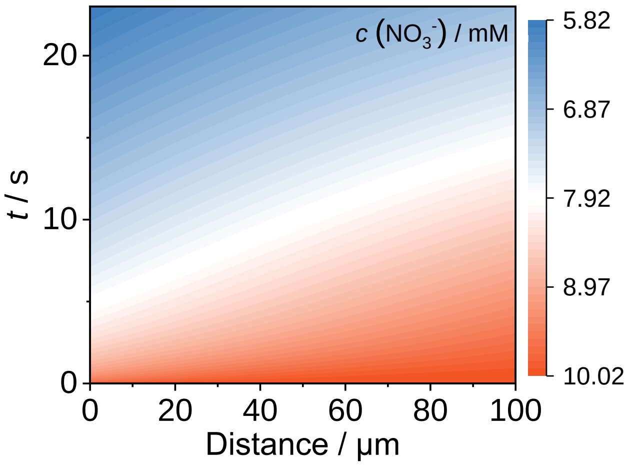

Supplementary Fig. 24 The FEA simulated NO_3^- distribution at the cathode-solution interface under potentiostatic conditions with an initial nitrate concentration of 10 mM.

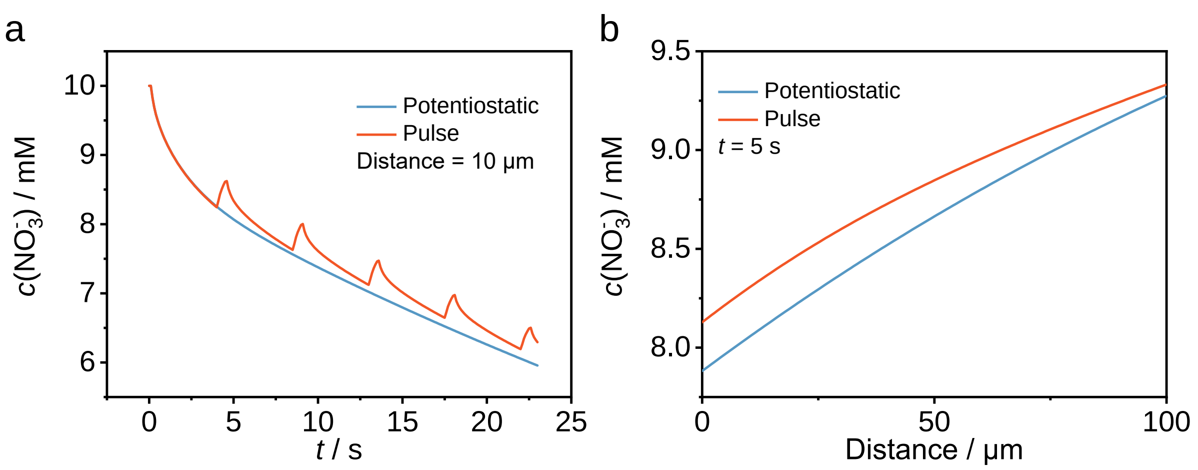

Supplementary Fig. 25 Nitrate concentration profiles simulated by FEA. (a) Time-dependent nitrate concentration distribution at a distance of 10 μm from the cathode surface and (b) distance-dependent nitrate concentration distributions a t = 5s under a constant (E = -0.1 V) and pulsed voltages (E_c = -0.1 V, E_a = +0.6 V, t_c = 4 s, t_a= 0.5 s).

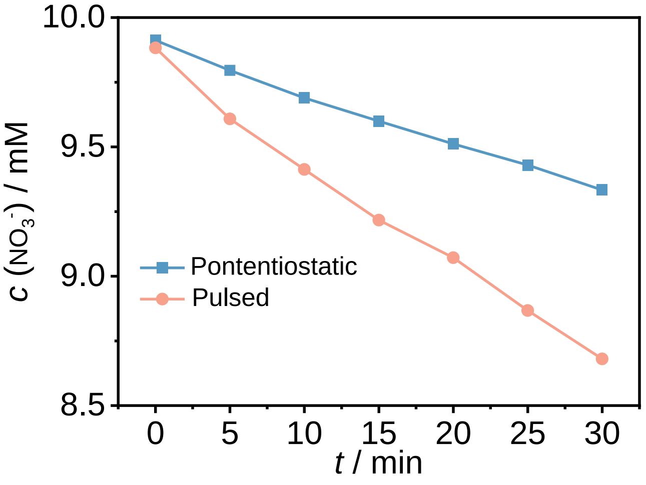

Supplementary Fig. 26 Time-dependent nitrate concentration changes under different conditions.

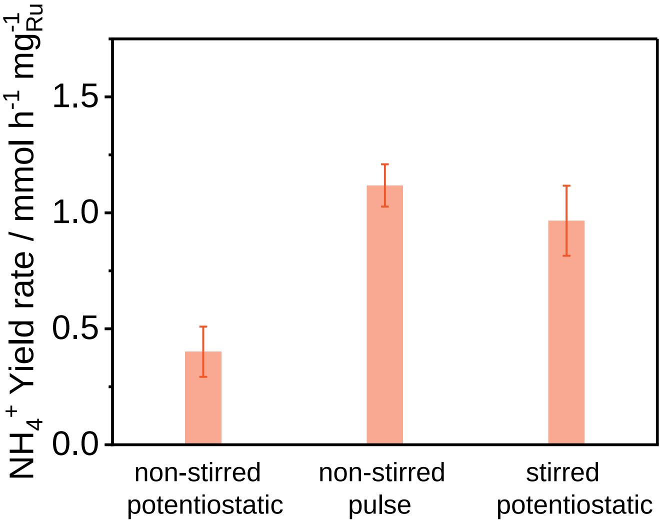

Supplementary Fig. 27 Ammonia yield rate over $RuIn_3/C$ obtained under different conditions using a rotating disk electrode. The rotating rate is kept at 1800 rpm under stirred conditions.

Compared with the non-stir potentiostatic condition, the ammonia yield rate increases by ~60% than at the stir potentiostatic condition (1800 rpm), indicating the existence of mass-transfer limitation for low-concentration nitrate electroreduction. Notably, the ammonia yield rate further increases under pulse conditions without stirring. These results prove that the alternated potential can not only weaken the mass-transfer limitation in low-concentration nitrate electroreduction (Fig. 4) but also promote the thermodynamics of NRA.

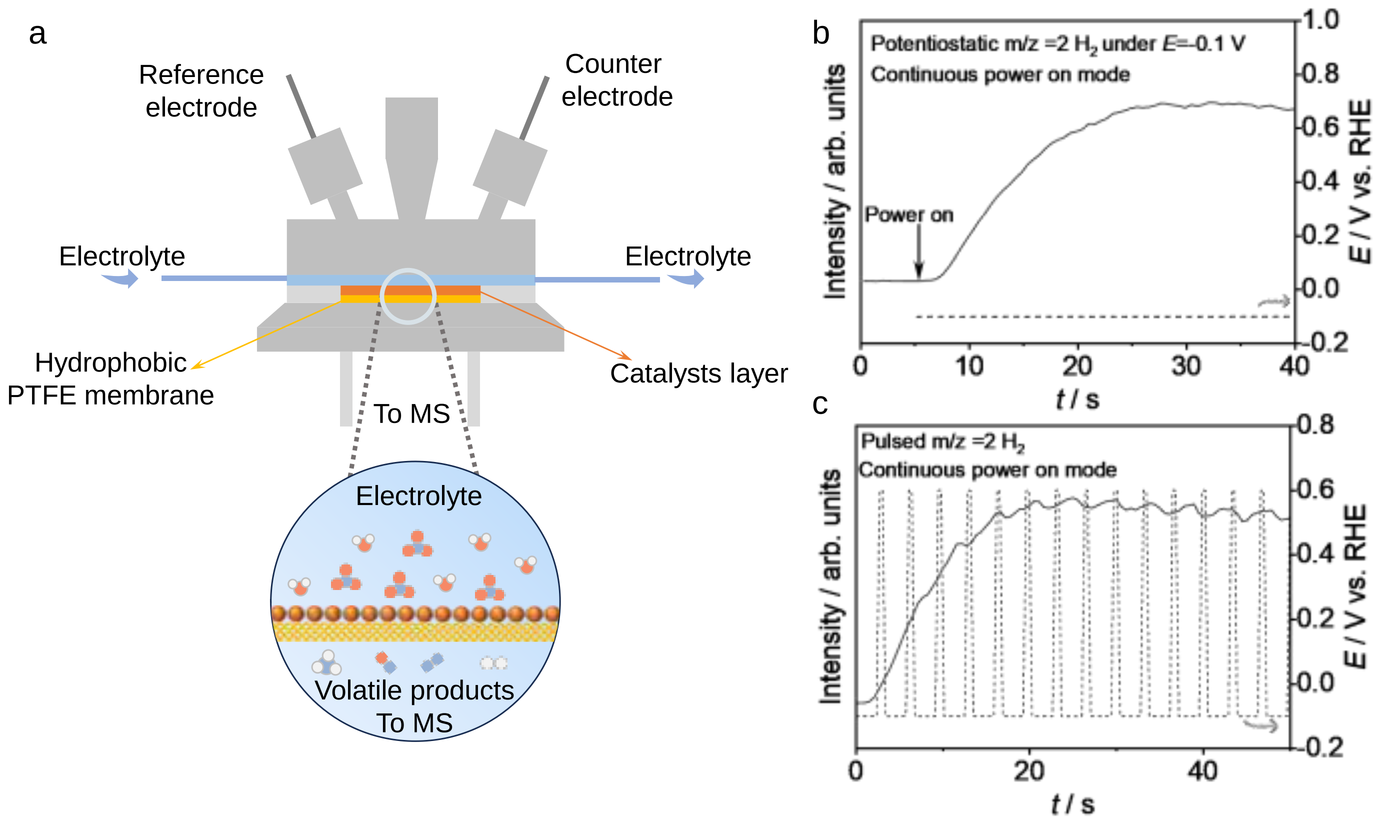

Supplementary Fig. 28 (a) Schematic illustration for the online DEMS electrochemical measurement. The enlarged signal intensity of m/z=2 under the application of continuous (b) constant potential (E = -0.1 V) and (c) pulsed potential (E_c = -0.1 V, E_a = +0.6 V, t_c = 4 s, t_a= 0.5 s).

For the online DEMS test, 0.1 M KOH containing 0.01 M NO_3^- keeps flowing through the homemade electrochemical cell through a peristaltic pump. The signal is gathered using a hydrophobic polytetrafluoroethylene (PTFE) membrane, essential for allowing volatile compounds while preventing water entry into the vacuum chamber. The produced volatile products are brought to the mass spectrometer through a pump (Supplementary Fig. 28a). The DEMS tests include two modes. The first mode is the application of continuous constant potential (E = -0.1 V) and pulsed potential (E_c = -0.1 V, E_a = +0.6 V, t_c = 4 s, t_a= 0.5 s) (Supplementary Figs. 28b, c). It can be seen that the H_2 signal first increases and then maintains well. The second mode is the alternative power switch on and off (Supplementary Fig. 29). The second mode includes three stages. In the first stage, the switch-on potential is constant (E = -0.1 V), and power switching on and off repeats 6 times. In the second stage, the switch-on potential is pulsed (E_c = -0.1 V, E_a = +0.6 V, t_c = 4 s, t_a= 0.5 s), and power switching on and off repeats 6 times. In the third stage, the switch-on potential is constant (E = -0.1 V), and power switching on and off repeats 6 times. The duration time for switch on and off are 50s and 180s, respectively. Thus, one switch-on period contains several pulse processes.

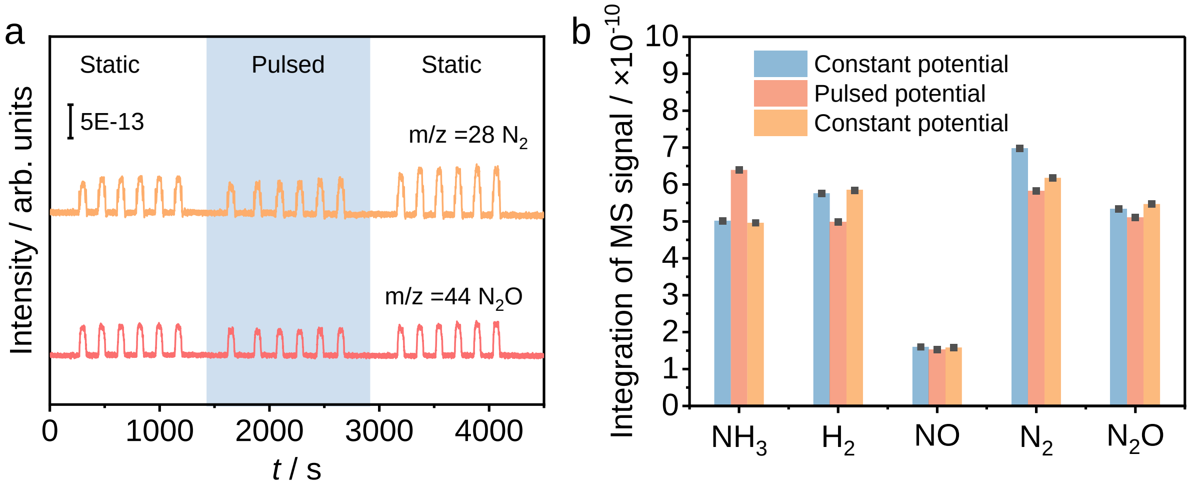

Supplementary Fig. 29 (a) Online DEMS signals of m/z=28 and m/z=44 during the NRA process. Starting with a constant potential (E = -0.1 V), switching to a pulsed potential (E_c = -0.1 V, E_a = +0.6 V, t_c = 4 s, t_a= 0.5 s), and finally switching back to the starting constant potential. (b) The corresponding DEMS signal intensities of NH_3, H_2, N_2, NO, and N_2O.

Except m/z signals of 2 (H_2), 17(NH_3), and 30 (NO), the m/z signals of 28 and 44, assigned to N_2 and N_2O, are detected by DEMS as well (Supplementary Fig. 29a). During the pulsed potential process, the signal intensities of H_2, N_2, NO, and N_2O shrink while the NH_3 intensity increases (Supplementary Fig. 29b). Moreover, the trend is the opposite when the power mode shifts back to a constant potential. Thus, the periodic change in signal intensities demonstrates the promotion of NH_3 generation and the suppression of H_2 during pulsed NRA.

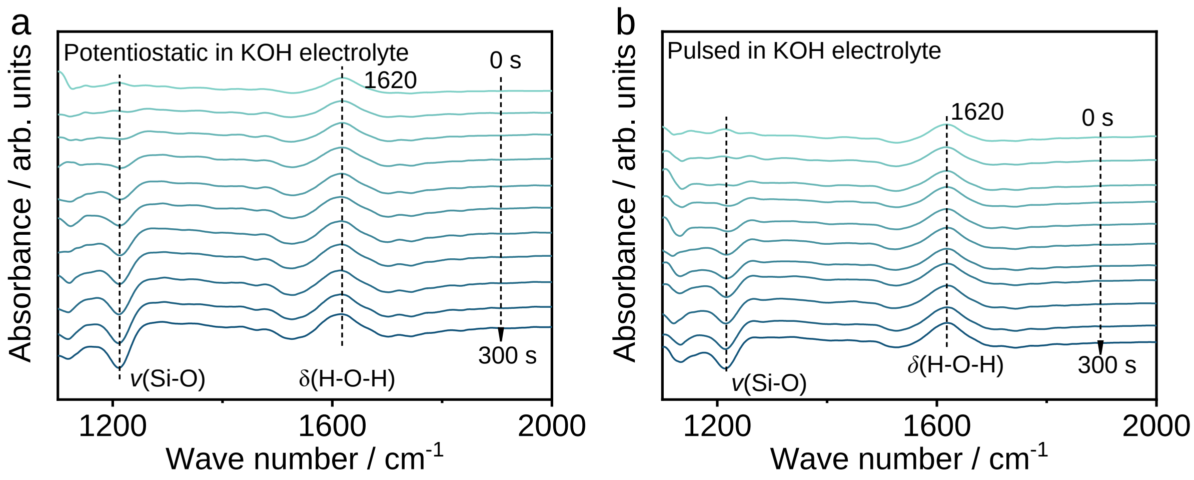

Supplementary Fig. 30. Time-dependent in situ ATR-FTIR spectra of NRA under (a) constant potential, (b) pulsed potential using 0.1 M KOH as electrolyte.

When using 0.1 M KOH solution as the electrolyte, only the band at 1620 cm^{-1}, attributed to the vibration of H-O-H[30], can be observed.

Supplementary Table 4. Constants used in the simulation.

**Parameters**	**Value**	**Description**
$c_0\_OH^-$	0.1 mol/L	Initial concentration of hydroxide
$c_0\_NO_3^-$	0.01 mol/L	Initial concentration of nitrate
$c_0\_K^+$	0.11 mol/L	Initial concentration of potassium
D_{K^+}	1.957×10^{-9} m^2/s	diffusion coefficient of potassium
$D_{NH_4^+}$	1.957×10^{-9} m^2/s	diffusion coefficient of ammonium
D_{OH^-}	5.273×10^{-9} m^2/s	diffusion coefficient of hydroxide
D_{H^+}	9.311×10^{-9} m^2/s	diffusion coefficient of proton
$D_{NO_3^-}$	1.902×10^{-9} m^2/s	diffusion coefficient of nitrate

Supplementary References

1. Chen, L.-W. et al. Intermetallic IrGa-IrO$_x$ core-shell electrocatalysts for oxygen evolution. *Nano Research* **15**, 1853-1860, (2021).

2. Rumble, J. *CRC handbook of chemistry and physics*. 97th edn, (CRC Press, 2017).

3. Casebolt, R., Levine, K., Suntivich, J. & Hanrath, T. Pulse check: Potential opportunities in pulsed electrochemical CO_2 reduction. *Joule* **5**, 1987-2026, (2021).

4. Ding, Y. et al. Pulsed electrocatalysis enables an efficient 2-electron oxygen reduction reaction for H_2O_2 production. *J. Mater. Chem. A* **9**, 15948-15954, (2021).

5. Kimura, K. W. et al. Controlled selectivity of CO_2 reduction on copper by pulsing the electrochemical potential. *ChemSusChem* **11**, 1781-1786, (2018).

6. Casebolt, R. et al. Effect of electrolyte composition and concentration on pulsed potential electrochemical CO_2 reduction. *ChemElectroChem* **8**, 681-688, (2021).

7. Timoshenko, J. et al. Steering the structure and selectivity of CO_2 electroreduction catalysts by potential pulses. *Nat. Catal.* **5**, 259-267, (2022).

8. Arán-Ais, R. M., Scholten, F., Kunze, S., Rizo, R. & Roldan Cuenya, B. The role of in situ generated morphological motifs and Cu(i) species in C_{2+} product selectivity during CO_2 pulsed electroreduction. *Nat.*

Energy **5**, 317-325, (2020).

9. Jeon, H. S. et al. Selectivity control of Cu nanocrystals in a gas-fed flow cell through CO_2 pulsed electroreduction. *J. Am. Chem. Soc.* **143**, 7578-7587, (2021).

10. Li, P. et al. Pulsed nitrate-to-ammonia electroreduction facilitated by tandem catalysis of nitrite intermediates. *J. Am. Chem. Soc.* **145**, 6471-6479, (2023).

11. Wang, Y. et al. Enhanced nitrate-to-ammonia activity on copper-nickel alloys via tuning of intermediate adsorption. *J. Am. Chem. Soc.* **142**, 5702-5708, (2020).

12. Niemann, V. A. et al. Co-designing electrocatalytic systems with separations to improve the sustainability of reactive nitrogen management. *ACS Catal.* **13**, 6268-6279, (2023).

13. Zhang, X. D. et al. Asymmetric low-frequency pulsed strategy enables ultralong CO_2 reduction stability and controllable product selectivity. *J. Am. Chem. Soc.* **145**, 2195-2206, (2023).

14. Han, S. et al. Ultralow overpotential nitrate reduction to ammonia via a three-step relay mechanism. *Nat. Catal.* **6**, 402-414, (2023).

15. Xu, Y. et al. Atomic defects in pothole-rich two-dimensional copper nanoplates triggering enhanced electrocatalytic selective nitrate-to-ammonia transformation. *J. Mater. Chem. A* **9**, 16411-16417, (2021).

16. Wang, Z. et al. Facile synthesis of carbon nanobelts decorated with Cu and Pd for nitrate electroreduction to ammonia. *ACS Appl. Mater. Interfaces* **14**, 30969-30978, (2022).

17. Sun, L. & Liu, B. Mesoporous PdN alloy nanocubes for efficient electrochemical nitrate reduction to ammonia. *Adv. Mater.* **35**, e2207305, (2022).

18. Song, Z. et al. Efficient electroreduction of nitrate into ammonia at ultralow concentrations via an enrichment effect. *Adv. Mater.* **34**, e2204306, (2022).

19. Sun, W. J. et al. Built-in electric field triggered interfacial accumulation effect for efficient nitrate removal at ultra-low concentration and electroreduction to ammonia. *Angew. Chem. Int. Ed.* **60**, 22933-22939, (2021).

20. Chen, G.-F. et al. Electrochemical reduction of nitrate to ammonia via direct eight-electron transfer using

a copper–molecular solid catalyst. *Nat. Energy* **5**, 605-613, (2020).

21. He, W. et al. Splicing the active phases of copper/cobalt-based catalysts achieves high-rate tandem electroreduction of nitrate to ammonia. *Nat. Commun.* **13**, 1129, (2022).

22. Chen, F.-Y. et al. Efficient conversion of low-concentration nitrate sources into ammonia on a Ru-dispersed Cu nanowire electrocatalyst. *Nat. Nanotechnol.* **17**, 759–767, (2022).

23. Li, J. et al. Atomically dispersed Fe atoms anchored on S and N-codoped carbon for efficient electrochemical denitrification. *Proc. Natl. Acad. Sci. U.S.A.* **118**, e2105628118, (2021).

24. Jia, R. R. et al. Boosting selective nitrate electroreduction to ammonium by constructing oxygen vacancies in TiO_2. *ACS Catal.* **10**, 3533-3540, (2020).

25. Guo, S. et al. Insights into nitrate reduction over indium-decorated palladium nanoparticle catalysts. *ACS Catal.* **8**, 503-515, (2017).

26. Ch. Y. Wang, Y. D., J. Pezoldt, B. Lu, Th. Kups, V. Cimalla, and O. Ambacher. Phase stabilization and phonon properties of single crystalline rhombohedral indium oxide. *Cryst. Growth Des.* **8**, 1257-1260, (2008).

27. Yu, D. et al. Metastable hexagonal In_2O_3 nanofibers templated from InOOH nanofibers under ambient pressure. *Adv. Funct. Mater.* **13**, 497-501, (2003).

28. Yu, A. et al. The surface processes on Ru/Pt(111) as probed by cyclic voltammetry and in situ surface-enhanced Raman spectroscopy. *ACS Sustainable Chem. Eng.* **10**, 14826-14834, (2022).

29. Bhaskar, S., Dobal, P. S., Majumder, S. B. & Katiyar, R. S. X-ray photoelectron spectroscopy and micro-Raman analysis of conductive RuO_2 thin films. *J. Appl. Phys.* **89**, 2987-2992, (2001).

30. Yao, Y., Wang, H., Yuan, X.-z., Li, H. & Shao, M. Electrochemical nitrogen reduction reaction on ruthenium. *ACS Energy Lett.* **4**, 1336-1341, (2019).
